# A randomised controlled trial of the efficacy of the ABCD Parenting Young Adolescents Program: rationale and methodology

**DOI:** 10.1186/1753-2000-4-22

**Published:** 2010-08-19

**Authors:** Kylie Burke, Leah Brennan, Sarah Roney

**Affiliations:** 1 Parenting Research Centre, 232 Victoria Parade, East Melbourne, Victoria, Australia; 2 School of Psychology & Psychiatry, Monash University, Wellington Road, Clayton, Victoria, Australia

## Abstract

**Background:**

The transition to adolescence is a time of increased vulnerability for risk taking and poor health, social and academic outcomes. Parents have an important role in protecting their children from these potential harms. While the effectiveness of parenting programs in reducing problem behavior has been demonstrated, it is not known if parenting programs that target families prior to the onset of significant behavioral difficulties in early adolescence (9-14 years) improve the wellbeing of adolescents and their parents. This paper describes the rationale and methodology of a randomised controlled trial testing the efficacy of a parenting program for the promotion of factors known to be associated with positive adolescent outcomes, such as positive parenting practices, parent-adolescent relationships and adolescent behavior.

**Methods/Design:**

One hundred and eighty parents were randomly allocated to an intervention or wait list control group. Parents in the intervention group participated in the ABCD Parenting Young Adolescents Program, a 6-session behavioral family intervention program which also incorporates acceptance-based strategies. Participants in the Wait List control group did not receive the intervention during a six month waiting period. The study was designed to comply with recommendations of the CONSORT statement. The primary outcome measures were reduction in parent-adolescent conflict and improvements in parent-adolescent relationships. Secondary outcomes included improvements in parent psychosocial wellbeing, parenting self-efficacy and perceived effectiveness, parent-adolescent communication and adolescent behavior.

**Conclusions:**

Despite the effectiveness of parenting programs in reducing child behavioral difficulties, very few parenting programs for preventing problems in adolescents have been described in the peer reviewed literature. This study will provide data which can be used to examine the efficacy of a universal parenting interventions for the promotion of protective factors associated with adolescent wellbeing and will add to the literature regarding the relationships between parent, parenting and adolescent factors.

**Trial Registration:**

Australian New Zealand Clinical Trials Registry ACTRN12609000194268.

## Background

Many critical life changes occur during the developmental period of transition from childhood to adolescence. The commencement of secondary school coincides with the numerous shifts in physical, social and cognitive functioning associated with development and puberty [1,2]. The majority of children manage the transition to adolescence without experiencing major problems. However, if not well managed adolescence can be a time when both the adolescent and their family experience significant difficulties such as increases in parent-adolescent conflict, adolescent mental health issues and the uptake of high risk behaviors such as the use of alcohol and other drugs or early or unsafe sexual practices [1,3-7].

Along with community, school and individual factors, research has demonstrated the major role that parents play in building resilience in children and promoting the successful transition from childhood to adulthood [8-12]. Broad contextual factors, such as parental psychopathology or substance disorders, marital conflict and divorce have been linked to negative child outcomes [1,13,14]. Negative parenting practices such as harsh or inconsistent parenting, poor parental monitoring and the lack of reasonable boundaries and limits around adolescent behavior, can also place adolescents at risk for adverse outcomes [7,9,15-19].

Conversely, experiencing feelings of love and respect, and having a warm relationship with at least one parent, are associated with positive adolescent outcomes such as better engagement in school, better academic outcomes for children and a reduction in the influence of the drinking patterns of peers [20-24]. Likewise, effective parental problem solving, as well as supervision and monitoring during childhood and early adolescence, delay or prevent risk taking behaviors such as initiation to alcohol and other drug use, and early commencement of sexual activity [15,17,24,25].

Combined, this research indicates that adolescence is a key time for the development of high risk behaviors and that parenting during early adolescence has an important impact on adolescent outcomes. Therefore, early adolescence is a time during which the introduction of effective prevention strategies is of paramount importance [15,26]. A specific focus on the parent-adolescent relationship may provide the mechanism for these prevention strategies. In particular, programs that provide parents with information and strategies on how to maintain or develop positive relationships and deal with difficult behaviors with their adolescent children may be useful [8,27,28].

Parenting programs, particularly those utilizing a social learning approach and incorporating behavior management training, can be effective in producing positive outcomes for both parents and their children [29-33]. The majority of parenting interventions target preadolescent children [30,34-37] with a large body of research showing that earlier intervention with parents (e.g., starting in infancy or early childhood) is more effective than later intervention (e.g., starting later in childhood)[38]. Such early childhood parenting programs are not expected to prevent all future difficulties. Adolescence brings with it a range of new developmental and social challenges that are not present or relevant when children are very young. Research has shown that for parenting programs to be effective they must be developmentally timed to be relevant to the parents needs [31]. For example, the need to develop and implement monitoring of adolescent behaviours such as dating, attendance at parties and alcohol and other drugs are not relevant to parents or their children prior to the transition to adolescence. Discipline strategies will also necessarily change during the adolescent period, with strategies that were effective with younger children (e.g., Time Out) no longer effective or developmentally desirable.

This increased recognition of the importance of the transition to adolescence, the potential protective role of parents, and the opportunity to prevent negative outcomes for adolescents [39] has led to the development of a number of parenting programs targeting young adolescents [19,40-43]. Programs for parents of adolescents have typically aimed to improve parenting practices and reduce adolescent risk taking. Early programs were developed for the treatment of distressed families, such as those with adolescents exhibiting antisocial, conduct and delinquent behaviors [39], or to address specific risky behaviors, such as drug misuse [40]. These programs have been demonstrated to result in improvements in child factors such as aggression, conduct disorders and substance misuse [44]. More recently universally targeted adolescent parenting programs have also been developed with the aim of promoting parenting practices that might facilitate prevention or early intervention for serious adolescent mental health, antisocial and delinquent behaviors [45,46].

Despite the increased recognition of the relevance of programs about raising adolescents, research in this area has tended to report lower levels of engagement and higher levels of drop out than programs targeting parents of young children [45-51] with participation rates of parents of older children as low as twenty to twenty-five per cent [52]. Factors that have been identified as impacting on parental enrolment and retention in parenting skill programs include the parents health beliefs (e.g., perceptions of severity of adolescent problems, perceived susceptibility to problems and potential benefits of the program); family socio-economic factors (such as parental education level) and family context (e.g., time demands, scheduling conflicts, timing and frequency of sessions)[53]. Given this, prevention efforts aimed at parents of older children need to incorporate strategies that have been shown to increase parental motivation and actual enrolment in parenting programs. For example, offering programs within close proximity to the family home, with sessions held no more than once per week and preferably on weeknights would address some of the potential contextual barriers to participation[53].

Further to difficulties with engagement and retention, evaluations of parenting programs suggest that traditional behavioral models of parenting interventions are insufficient to meet the needs of all families [54-56]. As such it may be useful to supplement these more established models of intervention with alternative theoretical models based on acceptance or mindfulness [54-58]. These adjunctive theoretical approaches may strengthen outcomes of behavioral family interventions by assisting parents to develop strategies for dealing with thoughts and emotions that may act as barriers to engagement and retention of parents of adolescents in parenting interventions and to the implementation of parenting strategies.

Further research is required to determine whether preventative parenting interventions delivered prior to the onset of difficulties are an effective method for changing family and other contextual factors sufficiently to improve the wellbeing of adolescents and their parents. It is also important to further investigate whether adjunctive theoretical approaches such as mindfulness or acceptance can improve engagement and retention and strengthen outcomes of interventions targeting parents of young adolescents. Therefore, the primary aim of the present research is to examine the efficacy of a parenting program that combines a behavioral family intervention approach with acceptance-based strategies in promoting improved parental wellbeing, parenting practices, parent-adolescent relationships and adolescent behaviour. A secondary aim of this research program is to add to the literature regarding the relationships between parent, parenting and adolescents factors.

## Method/Design

### Study Procedures

#### Study Design

This study was a randomised controlled trial in which 180 parents of adolescents were randomly allocated to one of two conditions; intervention or a wait-list control group. Parents in the intervention group participated in the ABCD Parenting Young Adolescents Program, a 6-session behavioral family intervention program which also incorporates acceptance-based strategies [57,59,60]. Those in the wait-list control group did not receive intervention during the 6-month wait list period. Assessments were conducted prior to intervention, immediately after intervention, and 6 months and 18 months after completion of intervention. The wait-list control group completed assessments at the same time as pre, post and 6-month follow-up assessments. The primary outcome measures were improvements in parental psychosocial well being, parenting practices and parent-adolescent relationships. Secondary outcomes included improvements in adolescent behavior and perceived parenting competence. The study was designed to be compliant with the recommendations of the CONSORT statement. [61,62]

#### Study Setting

The ABCD Parenting Program was delivered in community settings throughout the North and West Metropolitan Region of Melbourne, Australia. To maximize accessibility for parents, both daytime and evening sessions were offered and a range of venues, including primary and secondary schools, Community Health Centres, and Neighbourhood Houses.

#### Ethics Approval and Registration as Clinical Trial

The project received ethics approval from four separate ethical standard bodies; the Victorian Government, Department of Human Services Human Research Ethics Committee, RMIT University, the Victorian Department of Education and Training and the Catholic Education Office, Archdiocese of Melbourne Human Research Ethics Committees. The project has been registered as a clinical trial with the Australian New Zealand Clinical Trials Registry (ANZCTR) which sets the standards for the uniform reporting of the minimum registration data set as determined by the World Health Organization and the International Committee of Medical Journal Editors. ANZCTR Registration Number is ACTRN12609000194268.

#### Eligibility Criteria

Participants were included in the study if they met the following criteria; (a) parents of an adolescent aged 9-14 years, (b) custodial parents or non-custodial parents with regular access to their children, (c) English speaking, and (d) living in the north and west metropolitan region of Melbourne. Families were excluded from the study if (a) parents or adolescents had learning or developmental difficulties, (b) parents or adolescents had a mental illness, (c) adolescents had a medical condition or physical disability, or (d) adolescents were receiving other specialist services (e.g., Child and Adolescent Mental Health Services, private psychologist or psychiatrist) or participating in other research projects. These inclusion and exclusion criteria were assessed via parent self-report.

#### Recruitment

Information about the ABCD Parenting Program was circulated throughout the community via advertisement in local newspapers and Regional Parenting Centres' newsletters. Letters and promotional materials were also provided to schools, neighbourhood houses, General Practitioners, medical and community health centres and professionals registered with the Parenting Research Centre's professional mailing list. All recruitment information requested that parents contact the Parenting Research Centre for more information about the program. Professional referrals were only accepted after parents contacted the centre themselves.

#### Intake Process

Enquiries from parents living or working in the appropriate catchment area, with children aged 9 to 14 years of age were forwarded to the project team by centre administrative staff. A minimum of three attempts were made to contact these parents via telephone or email. Parents who could be contacted were first assessed against the eligibility criteria. Those who were not eligible were provided with other referral options. Eligible parents were given a detailed outline of the research including a review of the intervention program and assessment procedures and details of the randomisation, evaluation and wait-list processes. Those electing to proceed were asked to complete a telephone conducted intake survey. This survey was used to collect data about project inclusion and exclusion criteria, parenting experiences, and general demographic information.

Research assistants were trained to conduct these intake calls using a standardised script. The principal investigator monitored intake calls until research assistants demonstrated a sufficient level of competency. Random checks of intake calls were conducted throughout the project to ensure compliance with procedures. Following the intake call, eligible parents who indicated an interest in participating in the program were mailed a plain language statement, consent form and family demographic survey. Parents were asked to return the consent form and family demographic survey to register for the program.

Families who did not return this information were contacted to complete a telephone conducted survey regarding their reasons for non-registration. The research assistant conducting the survey classified responses under the following categories; other family/life issues, problem resolved, project wasn't explained clearly over the phone, written information sent out was unclear or overwhelming, not comfortable completing questionnaires, didn't want to wait, program wasn't appropriate to my needs, can't remember, or other. Multiple responses were possible and all responses identified by parents were recorded. Three attempts were made to complete the telephone conducted survey with each family. A self-report version of the survey, with an invitation to return the survey by mail, was provided to those families who could not be contacted via phone.

#### Allocation to Condition

Upon receipt of the consent form and family demographic survey parents were randomly allocated to the intervention or wait-list control condition using a web based computerised randomisation plan generator (http://www.randomisation.com). This program randomises each participant to a single treatment by using the method of randomly permuted blocks. A research assistant not involved in the delivery of the program, placed participants on the randomisation list in the next available slot. Parents were then contacted by telephone to inform them of this allocation. Those allocated to the intervention condition were booked into the next scheduled group on a day and time that suited the parent. Those in the wait-list condition were advised that they would receive a phone call one month prior to their commencement date to book them into a group that was being held on a day and time that suited them.

#### Group Formation

Parents were contacted by phone and enrolled into a group. Groups were scheduled to commence each school term for the state of Victoria, Australia. A variety of locations, days and times were available and parents selected the most convenient group. Groups were conducted with a minimum of 4 enrolments, and a maximum of 15 enrolled participants. All registered participants were sent reminder letters one to two weeks prior to the scheduled start date, and received a reminder call one day prior to the group commencement.

### Assessment

The efficacy of the ABCD Parenting Program was assessed using a range of self-report assessments. Both parents were able to attend the ABCD program, however, all families were asked to nominate a primary participant for the purposes of the research. This paper reports on the data provided by these primary participants only.

#### Measures

Parents were asked to complete a questionnaire battery which included measures of demographic information, parenting stress, parenting practices, parent psychopathology, parental monitoring, parent-adolescent conflict and adolescent behaviour.

#### Family Demographic Survey

The Family Demographic Survey was developed specifically for the purposes of this study. This instrument collects family demographic information including contact details, marital status, employment and education, family composition, health and development.

#### Strengths and Difficulties Questionnaire

The Strengths and Difficulties Questionnaire [63,64] was used as a measure of parental perception of their adolescent's prosocial and difficult behaviours. It includes 25 items, rated on a 3-point Likert scale, measuring the frequency of positive and negative behaviours. The measure provides a Total Difficulties score and 5 subscale scores; Emotional Symptoms, Conduct Problems, Inattention/Hyperactivity, Peer Problems, and Prosocial Behaviour. The SDQ is available in over forty languages and has been found to have good concurrent validity and adequate reliability with Cronbach's alpha's ranging from.76 (Total Score) to.51 for Peer Problems [65]. The measure also has adequate discriminant and predictive validity [66].

#### Issues Checklist (ICL)

The Issues Checklist, [67] was included as a measure of the frequency and intensity of issues discussed by parents and adolescents. This questionnaire lists 44 common parent-adolescent issues. Parents are required to indicate if each item was discussed, how often, and how 'hot' (on a scale of '1 calm' to '5 angry') the discussion was regarding the item. The measure provides Frequency and Intensity of conflict scores. The Issues Checklist has adequate psychometric properties with high internal consistency (mothers α = .89 and.85 for fathers, [68] and adequate to good test - retest reliability (mothers α = .63 - .74; fathers α = .73 - .80; (Robin & Foster 1989).

#### Stress Index for Parents of Adolescents (SIPA)

The Stress in Parenting Adolescents Scale [69] was included as a measure of parental stress. It allows for the examination of the numerous domains of parenting stress including adolescent characteristics, parent characteristics, adolescent-parent relationship characteristics, life events and emotional stress, as well as an overall composite score for total stress. Ninety items on this 112-item scale require parents to indicate the degree to which they agree with each statement on a 5-point scale from Strongly Disagree to Strongly Agree. The 22 items on the stressful life events domain require parents to indicate whether each event has occurred in the previous 12 months. The SIPA has adequate psychometric properties with all subscales exhibiting high internal consistency (range α = .81 - .97) and adequate to good test - retest reliability (range α = .74 - .93). The SIPA also has established content, convergent and discriminant validity (Sheras et al.).

#### Depression Anxiety Stress Scale (DASS 21)

The Depression sub-scale of the short version of the Depression Anxiety Stress Scale, [70] was included as a measure of the symptoms of depression. This 7-item factor requires respondents to indicate how much each item applies to them on a scale of '0 Did not apply to me at all' to '3 Applied to me very much or most of the time. The depression factor has good internal consistency (α = .81, [71].

#### Alabama Parenting Questionnaire

The Alabama Parenting Questionnaire [72] was included as a measure of parenting practices. Four of the six sub-scales; Involvement, Positive Parenting, Inconsistent Discipline, and Other Discipline Practices, were included in the current study resulting in a 29-item scale. Each item refers to a parenting practice and respondents are required to indicate how often they typically use each of these practices on a 5 item scale ranging from Never to Always. The Alabama has adequate psychometric properties with included subscales exhibiting acceptable internal consistency across sub-scales (range α = .67 to .80) and established discriminant validity [72].

#### Authoritative Parenting Questionnaire - Parent report form

The Authoritative Parenting Questionnaire [23] is a measure of parenting style adapted from an adolescent report questionnaire, the "Authoritative Parenting Questionnaire" [73]. This 22 item parent report measure contains three factors: Involvement/Acceptance, Strictness/Supervision and Autonomy Granting. The parent report version of the measure has demonstrated acceptable reliability for the involvement (α = .80), autonomy granting (α = .74) scale and the strictness scales (α = 58) [23]. The original adolescent report measure by Steinberg et al (1992) has also demonstrated acceptable internal consistency (α = .72 for Involvement/Acceptance, α = .76 for Strictness/Supervision and α = .82 for Autonomy Granting).

#### Monitoring/Supervision Scale

The Monitoring/Supervision Scale (MSS, Parenting Research Centre, 2005) is a 5-item scale designed to assess importance, expectations and monitoring strategies reported by parents. The scale has adequate internal consistency (α = .60).

#### Consumer Satisfaction Scale

The Consumer Satisfaction Scale (CSS, Parenting Research Centre 2005) is a measure of consumer satisfaction with parenting programs. This 20-item questionnaire assesses the quality of the service provided, how well the program met the parent's needs and changed behaviour, and whether the parent would recommend the program to others. Parents are also prompted to make general comments or suggestions about the program.

#### Assessment Schedule

The Family Demographics survey was administered at registration, and the Consumer Satisfaction Scale was completed at post intervention. All other measures were completed by participants in both conditions at pre, post and 6-month follow-up. Those in the intervention condition also completed measures at 18-month follow-up.

Pre-intervention assessments were posted to participants in the intervention condition two weeks prior to the commencement of their scheduled group. Parents were asked to return their questionnaires to the first group session. Post-intervention assessments were distributed in the final group session and parents were asked to return the questionnaire in the reply-paid envelop provided. The first follow up assessment was conducted 6 months after program completion. Questionnaires were mailed to parents with a request that they be returned in the reply-paid envelope provided.

Participants in the wait-list condition received pre-intervention, post-intervention and follow-up questionnaires at the same time as parents in the intervention condition. Questionnaires were mailed to parents with a request that they be returned in the reply-paid envelope provided. Generally, parents allocated to the wait-list condition completed their follow-up questionnaires just prior to participating in the ABCD Parenting Program.

Parents in the intervention condition also completed a long-term follow-up assessment 18 months after program completion. It was not possible to conduct long-term follow-up assessments with parents in the wait-list condition as it was not considered ethical to delay intervention for longer than 6 months.

#### Questionnaire Return Process

A number of steps were taken to promote maximum return rates. If questionnaires were not returned within 14 days of distribution, parents were contacted by phone. During the call, the research assistant answered any questions about the questionnaires and arranged for another to be sent if required. A second reminder call was made if questionnaires were not returned within 21 days of distribution. If questionnaires were not received within 28 days of distribution parents were sent a reminder letter, and a second copy of the questionnaire mailed to them.

### Intervention

*The ABCD Parenting Young Adolescents Program *(Cann, Burke & Burke, 2003): ABCD is a group program for parents of children aged from 9-14 years. The program is a brief psycho-educational intervention based on social learning principles and incorporating acceptance-based strategies. An emphasis is placed on active skills building and problem solving, and strategies that have been shown by research to reduce distress in interpersonal relationships. The aim of the program is to provide parents with information and skills for developing and maintaining trusting, positive and accepting relationships with their young adolescents which, in turn, encourages them to test their independence within safe boundaries.

The program is delivered over six consecutive weeks. During each 2-hour session parents have the opportunity to discuss, practice and receive feedback on a range of strategies and ideas. The program content is designed to enhance parental understanding and skills for assisting their children to make the transition to adolescence. Content is organised under four themes: 1) developing understanding and empathy for adolescents; 2) building strong relationships; 3) building responsibility and autonomy; and 4) parental self-care. An overview of program objectives and content is provided in Table 1.

**Table 1:** ABCD Parenting Young Adolescents Program Overview

Theme	Objective	Content
Developing understanding/empathy for adolescents	To provide participants with an understanding of the developmental and social challenges facing adolescents and their parents during the transition from childhood to adulthood. To increase parental empathy for their adolescent To assist parents to identify their core values relating to parenting and establish concrete actionable goals.	Understanding Adolescence (Session 1) Parenting Traps (Session 1) Identifying Family Values (Session 2)
Building strong relationships:	For participants to have a clear a clear understanding of the importance of establishing and maintaining a positive parent-adolescent relationship. For participants to practice a range of strategies for building and enhancing their skills for connecting and communicating with their adolescents.	Connecting (Session 2) Positive Feedback (Session 2) Communicating (Session 3)
Building adolescent responsibility and autonomy	For parents to be able to effectively use a model for establishing boundaries around their own and their adolescent's behaviour. To provide parents with skills for dealing with problems arising with their adolescent. To provide parents with strategies for backing up agreements and/or parent decisions. To assist parents to identify, prevent and/or manage potential risk-related problems facing their adolescent.	Boundaries (Session 4) Problem Solving (Session 4 & 5) Setting Limits (Session 5) Monitoring (Session 5) Problem Solving and Risk Taking (Session 6)
Parental Self-Care	For parents to develop a range of options for maintaining their own well-being. To provide parents with a range of information regarding seeking help.	Dealing with Strong Emotions (Session 5 & 6) Self Care (Session 6) Getting Support (Session 6)

#### Facilitator Training

Five group facilitators were trained by the first author, one of the program developers, thus a total of 6 facilitator's delivered programs as part of the project. The level of training was matched to the needs of the facilitator so that less experienced trainers received more comprehensive training in delivering group interventions, conducting parenting training, responding to group processes issues and content specific to the ABCD Parenting Program. Facilitators with more experience in delivering group based parenting interventions received training in the ABCD Parenting Program theory and content only. Prior to delivering groups independently, all facilitators co-facilitated groups with the first author until they demonstrated competence in delivery of the program. All facilitators received weekly supervision from the first author throughout the project. Audiovisual footage of group sessions was used throughout these supervision sessions to allow for the provision of feedback on both group process and program content.

#### Group Materials

The ABCD Parenting Program was delivered as prescribed in the ABCD Parenting Young Adolescent Facilitators Manual [74]. The manual contains detailed session notes for facilitators and PowerPoint slides and parent handouts for use during the program.

#### Treatment Adherence

Treatment fidelity and integrity was maintained across facilitators via the use of a manualised program [74]. Facilitators completed adherence checklists at the end of each group session. All group sessions were videotaped and segments of each session were randomly selected and reviewed in supervision sessions to ensure adherence to program content and process.

#### Statistical Power

As there were no meta-analyses exploring outcomes of adolescent parenting programs available at the time of the study, results of a meta-analysis exploring the effectiveness of behavioural parenting training in modifying antisocial behaviour in children was used for power analyses[75,76]. This paper demonstrated a medium to large effect size for child behaviour outcome measures (d = 0.84) and a small to medium effect size for parental adjustment outcome measures (d = .44) [77]. G-Power [78,79] was used to calculate the required sample size. This analysis indicated that for power = 0.80 with an alpha = 0.01, 21 participants per group would be required for child outcome variables, and 64 participants per group would be required for parental adjustment variables. Given the expected high rate of drop-outs and loss to follow-up for participants in the study, particularly those on the wait-list, the number of participants recruited to the experimental and wait-list groups was increased to 90, ensuring that the required sample size was achieved.

#### Planned Statistical Analysis

Data will be analysed using SPSS. Analysis will be preceded by data cleaning and assumption testing. A series of analyses are planned. The primary analyses will assess the efficacy of the ABCD Parenting Program in promoting improved parental wellbeing, parenting practices, parent-adolescent relationships and adolescent behaviour. Intervention efficacy will be assessed by comparing the outcomes of the wait-list control and intervention conditions post intervention and follow-up measures using a series of 2 × 2 mixed factorial Mancovas. Both completer and intention-to-treat analyses will be conducted. The between subjects factor will be condition (intervention, wait-list) and the within subjects factor will be time (post, follow-up). The maintenance of intervention effects will also be assessed by comparing pre, post and follow-up and long-term follow-up using repeated measures Manovas. Potential confounds (e.g., socioeconomic status) and moderators (e.g., child gender) will be explored.

Further analyses will include exploration of the predictors of intervention outcome, and a thorough analysis of treatment adherence, compliance with intervention and other process variables. Detailed descriptive analysis of pre-intervention adolescent, parent and family characteristics will also be conducted to thoroughly explore the characteristics of families attending an adolescent focused, universal parenting program. Secondary analysis will explore the relationships between parent, parenting and adolescents factors. Based on existing literature it is predicted that positive parenting practices and parental coping and wellbeing will be associated with positive adolescent outcomes. Structural equation modeling will be used to explore the direction and strength of these complex relationships.

## Results

### Recruitment

Participant recruitment commenced in January 2005 and was completed in February 2007, at which time 180 families had enrolled to participate, ensuring an adequate number of participants for statistical comparison. Recruitment drives were conducted at the commencement of each school term. The first ABCD Parenting Program group commenced in May 2005 and the final intervention group commenced in February 2007. Twenty-one ABCD Parenting Program groups were conducted throughout this period.

A total of 409 parents were contacted to complete an intake call. Twenty-one families (5%) were excluded from the study as they did not meet the eligibility criteria for one or more reasons. Of these, eight adolescents were receiving other specialist services, eight adolescents had a medical condition or physical disability, seven adolescents and four parents had a mental illness, four adolescents had learning or developmental difficulties, one was a non-custodial parent without regular access to their children, and one was non-English speaking. Thirteen (3%) parents chose not to continue with the intake call after receiving verbal information about the ABCD Parenting Program and the associated research.

Three hundred and seventy five eligible (92%) parents completed intake and were mailed a plain language statement, consent form and family demographic survey. The majority of intake calls were completed with the child's mother (79.9%), 42.1% of target children were female, and the age of target children ranged from 9 to 14 years (M = 11.81, SD = 1.41). One hundred and eighty parents returned their consent form and family demographic survey to register for the program.

One hundred and ninety-five (52%) parents who completed intake did not register for the program. Reasons for non-registration included; other family/life issues (36.1%), problem resolved (18.1%), verbal project explanation unclear (10.8%), written project information unclear (19.5%), not comfortable completing questionnaires (14.5%), didn't want to wait (13.3%), program not appropriate for needs (13.4%), can't remember (6.0%), and other (75.0%). Many families provided multiple reasons for non-registration.

Parents that did not register for the program did not differ from those that did register in terms of their adolescent's age (*t*(353.9) = -1.23, *p *> .05) or sex (*χ*^2^(1) = .64, *p *> .05). Parents that registered for the program(*M *= 1039.6, *SD *= 68.1) had a higher Socioeconomic Indexes for Areas(SEIFA) Index of Relative Socio-economic Advantage and Disadvantage based on residential postcode(*t*(342.9) = 2.39, *p *= .017) than those who did not register for the program(*M *= 1021.5, *SD *= 74.6). This indicates that families of lower socioeconomic status were less likely to register for the program.

**Figure 1:**
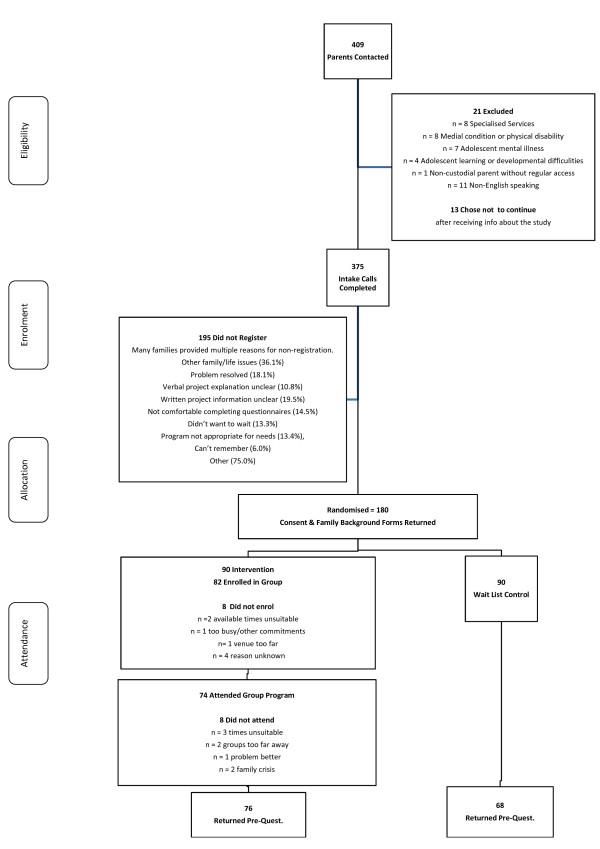
**Recruitment process from enquiry to group allocation return of pre-questionnaire**.

The 180 participants who returned consent forms and family demographic surveys were randomly allocated to intervention (n = 90) or wait-list control (n = 90) conditions. Of those allocated to the intervention condition 82 (91%) were successfully enrolled into a group. It was not possible to enrol 8 (9%) registered parents in the intervention condition into groups. Eight (10%) of the 82 enrolled in a group did not attend. Of the 90 parents allocated to the intervention condition 76 (84%) returned a completed pre-questionnaire. This included all 74 parents who attended a group and two parents who were enrolled in a group but did not attend. Sixty-eight (76%) of the 90 parents allocated to the wait-list condition returned a completed pre-questionnaire. This information is presented in Figure 1 below.

### Participant Characteristics

The sample comprised 160 mothers, 19 fathers and 1 step-father of young adolescents aged from 9.0 to14.6 years (*M *= 11.82, *SD *= 1.40), 52.2% of which were male. Where Australian 2006 population demographic data [80], [81] for parents of a similar age to the current sample are available they are summarised and compared in the tables 2, 3 and 4.

**Table 2:** Current Sample and Australian Population Data for Country of Birth, Government Benefits and Family Circumstances

	Current Sample	Australia (2006)
Australian Born	73%	77%
Government Benefits	27%	21%
Family Circumstances		
Original 2 Parent(%)	56	72
Step 2 Parent(%)	12	7
Sole Parent(%)	30	22
Other(%)	3	-

**Table 3:** Current Sample and Australian Population Data for Highest Education and Mental Health Professional Help Sought

	Current Sample		Australia (2006)
	Participant	Partner	
Highest Education			
Post-Graduate(%)	25	30	4
Undergraduate(%)	27	23	28
TAFE/Trade(%)	18	20	26
High School(%)	29	27	40
Mental Health Professional			
Psychologist(%)	9	4	7
Psychiatrist(%)	3	1	8
Counsellor(%)	12	3	-
Social Worker(%)	4	2	-
Other Professional(%)	2	1	2.4

Seventy-three percent of participating parents were Australian born. Fifty-six percent of participants were living in a two parent original family, with 12 % in a step-family, and 30% in a sole parent family and 3% in other family types. Twenty-seven percent of participating families received a government benefit or pension, 43% had received other parenting education in the past 2-years and 15% of target children had regular contact with another professional or government agency for emotional or behavioural problems. Of note, while all parents included in the study indicated that their adolescent was not accessing specialised services at intake, a high proportion of these parents went on to acknowledge receipt of specialised services on the demographic survey. Comparisons to Australian population data are summarised in Table 2.

Twenty-five percent of participating parents had post-graduate qualifications, 27% undergraduate qualifications, 18% TAFE/Trade qualifications, 13% had finished secondary school, and 16% indicated that they had not completed high school. Similarly, thirty percent of partners had post-graduate qualifications, 23% undergraduate qualifications, 20% TAFE/Trade qualifications, 11% completed secondary school and 17% indicated that they had not completed high school. Comparisons to Australian population data are summarised in Table 3.

Thirty percent of participating parents had received professional help in the past 6-months. Nine percent had received help from a psychologist, 3% from a psychiatrist, 12% from a counsellor, 4% from a social worker, and 2% from another professional. Only 9% of partners had received professional help in the past 6-months. Four percent had received help from a psychologist, 1% from a psychiatrist, 3% counsellor, 2% from a social worker, and 1% from another professional. Comparisons to Australian population data are summarised in Table 3.

Of the 160 mothers completing questionnaires, 19% were employed full-time, 51% were employed part-time and 31% were not in paid employment. Of the 111 male partners 87% were employed full-time, 5% were employed part-time, and 9% were not in paid employment. Of the 20 fathers who completed questionnaires, 80% were employed full-time, and 20% were not in paid employment. Of the 14 female partners, 36% were in full-time paid employment, 43% worked part-time, and 21% were not in paid employment. Comparisons to Australian population data are summarised in Table 4.

**Table 4:** Current Sample and Australian Population Employment Data

	Mothers		Fathers	
	Current Sample	Australia (2006)	Current Sample	Australia (2006)
Participating Mothers	(n = 160)		(n = 111)	
				
Full Time Employed(%)	19	25	87	85
Part Time Employed(%)	51	37	5	6
Not in Paid Employ. (%)	31	38	9	9
				
Participating Fathers	(n = 14)		(n = 20)	
				
Full Time Employed(%)	36	25	80	85
Part Time Employed(%)	43	37	-	6
Not in Paid Employ. (%)	21	38	20	9

## Discussion

This paper describes the rationale and design of a randomised controlled trial testing the efficacy of a behavioural family intervention program incorporating acceptance-based strategies. A secondary aim of this research program is to add to the literature regarding the relationships between parent, parenting and adolescents factors.

### Recruitment and Participation

This study involved the delivery of the ABCD program to parents within community settings. Analysis of the extensive recruitment and enrolment process undertaken for the study reveals some interesting findings. First, the large number of parents expressing interest in the program indicates that programs for parents of young adolescents are both appealing and sought after by parents during the transition to adolescence. Secondly, this interest translates into registration, as a large proportion of parents who completed an intake survey, and who were eligible to participate, went on to register for this study.

The predominant reasons given for non-registration were factors associated with the family or adolescent or to the demands associated with participating in a research project (e.g., not wanting to complete questionnaires or wait for intervention). These results illustrate the importance of incorporating strategies to engage families during the early stages of recruitment. Additionally, it is important to consider methods for reducing the demands of research processes on participants, (e.g., simplifying plain language descriptions and consent procedures).

### Participant Characteristics

Analysis of the characteristics of participants allocated to the intervention or wait-list conditions showed a number of features of the sample worth noting, including some important differences between study participants and Australian population norms. The study sample comprised a majority of mothers with only 11% of primary participants being fathers. A key difference between the current sample and Australian population norms related to family circumstances. This sample had a higher representation of sole and step families and a lower number of original, two parent families than recorded in the 2006 Australian Census [80]. These results indicate that the participants in this research program have a higher proportion of family arrangement linked to vulnerability than reported in the general Australian population.

The participants in this study also reported higher levels of post-graduate qualifications and lower levels of TAFE and high school qualifications than those reported in the 2006 Australian Census. However, these results are consistent with Spoth [82] who reported that parents with a higher level of education are more likely to enroll in parenting programs. Reported employment rates show a high proportion of part-time employed mothers with partners in full-time employment compared to Australian population norms. Attending fathers were more likely to be full time employed or not in paid employment and were less likely to be working part-time than the Australian population norms. These results indicate that the program is particularly attractive to highly educated parents who were not in full-time employment and to families having experienced separation or relationship breakdown. Similarly, of the parents who called to inquire about the program, families of lower socioeconomic status were less likely to go on to register for the program. This finding is consistent with previous research indicating that families of lower socioeconomic status perceive more barrier to treatment participation [83]. The additional demands associated with the research requirements of this study likely further exacerbated this problem.

Reports of help seeking are also noteworthy. Almost half of the participants in the current study had sought assistance from professionals in the past two years, with a proportion having also sought assistance for problems with their child in the past. More than half of the participants reported that they had not previously sought support. These results indicate that whilst a significant proportion of participants were not currently reporting difficulty, a large subset of the participants had in the past experienced some difficulty with their parenting or children. This suggests that the sample is not purely a preventative one but may include a subset of families that require treatment services. Such a finding is particularly noteworthy given the universal recruitment methods adopted for the study. Preventative interventions have been classified as falling into three potential categories, universal, selective or indicated ([84]. Mrazek and Haggerty [84] defined universal prevention as interventions targeted for the general population or to a population group that has been specified due to increased risk, with selected and indicated interventions targeting increasingly specific levels of risk in subgroups of the population. The current study adopted a universal approach, whereby families were eligible to participate on the basis that the period of adolescence represents increased vulnerability to risk. The past help seeking reported by the current sample is not unexpected in light of the universal recruitment approach. In any general population one would expect to find such a cross-representation of participants, from those not experiencing difficulty to those who have or are experiencing low or subclinical levels of difficulty.

## Strengths and Limitations

This study has been designed to address many of the limitations of previous research and to comply with the recommendations of the CONSORT statement [61,62]. The intervention was informed by theory and empirical research and directly targets the improvement of parenting skills, parental wellbeing and parent-adolescent relationships, factors known to protect against negative adolescent outcomes. Participating parents were randomly allocated to intervention or wait-list control conditions. A range of parent, parenting, adolescent and family outcomes were assessed using measures published in peer reviewed publications and validated for the present purpose. The long-term follow up for the intervention group is a particular strength of the present study. Follow-up at 6 month and 18 month intervals of the study will allow for the assessment of maintenance of change, and the efficacy of the program as an effective prevention strategy for promotion of factors known to protect adolescent from negative health and behavioural outcomes. The CONSORT tracking method [61,62] will also be used to allow for consideration of engagement processes and a thorough assessment of intervention attrition. This will also allow for both intention-to-treat and completer analyses.

While the design of the current study addresses many of the limitations of previous research, several methodological issues are acknowledged. As the research was conducted within a clinical service it was necessary to offer participants allocated to the wait-list control condition intervention thus comparison between groups is not possible during the longer term follow-up period. Additionally, as there are few measures designed for assessment of impact of adolescent prevention programs, the measures used in the current study were originally designed for assessment of the impact of treatment programs. Consequently, these measures may not be sensitive enough to detect the impact of a prevention program. Further, as a result of the reliance on self-report measures this study includes only subjective reports of outcomes. The inclusion criteria used in the present study were necessary to ensure internal validity, however they do impact on the external validity of the study and hence the generalisation of results. For example non-English speaking families were not included in this study and thus result may not apply to this important group. Similarly, the self-selected sample used in this study was relatively well educated and of high socioeconomic status, consequently, results may not apply to families with lower levels of income and/or education.

Despite these limitations the current study will provide much needed information about the efficacy of parenting interventions for families with young adolescent children.

## Conclusion

In conclusion, the present study will provide much needed information about the role of parents in the promotion of factors that prevent serious psychological and behavioral difficulties for young people. Outcome analysis will assess the impact of a behavioral family intervention program incorporating acceptance-based strategies on parenting practices, parent-adolescent relationships, and adolescent outcomes. It will also explore the relationships between parent, parenting and adolescents factors.

## Competing interests

The ABCD Parenting Program was co-authored by KB. The Parenting Research Centre has the rights to this program and all authors were employed by the Parenting Research Centre during design and implementation of the trial and development of the manuscript.

## Authors' contributions

KB co-authored the ABCD parenting program, trained and supervised all facilitators, facilitated groups, designed and managed the efficacy trial, and contributed to manuscript development. LB facilitated groups, designed and assisted with the management of the efficacy trial, was responsible for data cleaning and analysis, and contributed to manuscript development. SR assisted with participant and data management. All authors have read and approved the final manuscript.
